# Peroxydisulfate-assisted sonocatalytic degradation of metribuzin by La-doped ZnFe layered double hydroxide

**DOI:** 10.1016/j.ultsonch.2022.106236

**Published:** 2022-11-23

**Authors:** Sultan Akdağ, Tannaz Sadeghi Rad, Ramazan Keyikoğlu, Yasin Orooji, Yeojoon Yoon, Alireza Khataee

**Affiliations:** a Department of Environmental Engineering, Faculty of Engineering, Gebze Technical University, 41400 Gebze, Turkey; b Department of Environmental Engineering, Faculty of Engineering and Natural Sciences, Bursa Technical University, 16310 Bursa, Turkey; c College of Geography and Environmental Sciences, Zhejiang Normal University, Jinhua 321004, China; d Department of Environmental and Energy Engineering, Yonsei University, Wonju, South Korea; e Research Laboratory of Advanced Water and Wastewater Treatment Processes, Department of Applied Chemistry, Faculty of Chemistry, University of Tabriz, 51666−16471 Tabriz, Iran

**Keywords:** Herbicide degradation, Hydrotalcite, Peroxydisulfate activation, Ultrasound process, Water treatment

## Abstract

Metribuzin is an herbicide that easily contaminates ground and surface water. Herein, La-doped ZnFe layered double hydroxide (LDH) was synthesized for the first time and used for the degradation of metribuzin via ultrasonic (US) assisted peroxydisulfate (PDS) activation. The synthesized LDH had a lamellar structure, an average thickness of 26 nm, and showed mesoporous characteristics, including specific surface area 110.93 m^2^ g^−1^, pore volume 0.27 cm^3^ g^−1^, and pore diameter 9.67 nm. The degradation efficiency of the US/La-doped ZnFe LDH/PDS process (79.1 %) was much greater than those of the sole processes, and the synergy factor was calculated as 3.73. The impact of the reactive species on the sonocatalytic process was evaluated using different scavengers. After four consecutive cycles, 10.8 % loss occurred in the sonocatalytic activity of the La-doped LDH. Moreover, the efficiency of the US/La-doped LDH/PDS process was studied with respect to the degradation of metribuzin in a wastewater matrix. According to GC–MS analysis, six by-products were detected during the degradation of metribuzin. Our results indicate that the US/La-doped ZnFe LDH/PDS process has great potential for efficient degradation of metribuzin-contaminated water and wastewater.

## Highlights

• Sonocatalysis is an effective option for the remediation of metribuzin.
• ZnFe LDH was doped with lanthanum to improve its sonocatalytic properties.
• Ultrasound/La-doped ZnFe LDH/Peroxydisulfate has a high synergistic effect.
• SO_4_^•−^ radicals played a major role in the sonocatalytic degradation of metribuzin.

## Graphical abstract

**Figure:**
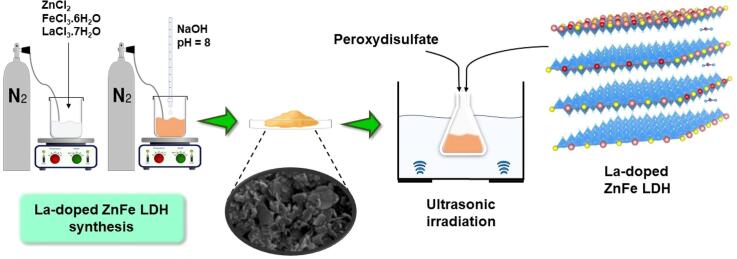

## 1 Introduction

Metribuzin is an asymmetrical triazine herbicide used to control the growth of grasses and broad-leaved weeds among crops such as tomatoes, potatoes, soybeans, sugarcane, turf grasses, and cereals [1]. Its mechanism of action is based on the inhibition of photosynthesis by impeding electron transport in the Hill reaction of photosystem II [2]. Owing to its high solubility in water (1050 mg L^−1^ at 20 °C), low octanol–water partition coefficient (K_ow_ = 44.7), and soil organic carbon partition coefficient (K_oc_ = 3.14–81.5 mL g^−1^), it can easily find its way into surface and groundwater [1]. Metribuzin is moderately hazardous to fish and invertebrates, but it is very toxic to freshwater macrophytes and algae, and it is typically more harmful to aquatic plants than other commonly used herbicides [2]. Additionally, it may cause liver damage in mice at high dosages of 200 mg kg^−1^ [3]. Various water treatment processes, including adsorption [4], chemical oxidation [5], and advanced oxidation processes (AOPs) [6] have been developed to treat water containing metribuzin. AOPs are effective and environmentally-friendly methods for converting target pollutants into carbon dioxide (CO_2_) and water (H_2_O) by generating reactive oxygen species (ROS) [7]. Sulfate radical-based AOPs (SR-AOPs) are preferred over other AOPs because the sulfate radical (SO_4_^•−^) has a long lifetime (30–40 µs), high oxidation potential (E^0^ = 2.5–3.1 V), and wide pH (2–8) applicability [8]. Sulfate radicals are generated by the activation of peroxydisulfate (PDS) by light irradiation, ultrasonic waves, microwaves, transition metals, and heat, among others [9].

Sonolysis, a type of AOPs, is the formation, growth, and collapse of microbubbles, which produces localized hot spots with high temperature (5000 K) and pressure (1800 atm). Hot spots contain water that is thermally decomposed, and hence, consist of highly reactive radicals, such as H^•^ and ^•^OH, which can initiate oxidation reactions [10]. However, sonolysis is thought to be relatively lengthy and energy-consuming. To overcome these limitations, sonocatalysis, which is based on the synergistic effects of ultrasound and semiconductor catalysts, has been proposed [11]. In the sonocatalytic process, ultrasonic cavitation creates sonoluminescence (SL), which results in the emission of light over a broad wavelength range [12]. When a semiconductor metal is excited by SL, electrons pass from the valence band (VB) to the conduction band (CB), resulting in the formation of electron-hole pairs on the surface of the catalyst [13]. In addition, the number of active sites and reactive species increases owing to the activation of semiconductor catalysts by US irradiation during sonocatalysis [14]. Various semiconductors have been employed as sonocatalysts, including TiO_2_ [15], CaMoO_4_ [13], ZnWO_4_ [16], MgFe_2_O_4_ [17], and layered double hydroxides (LDHs) [18]. Among these, LDHs are considered advantageous, owing to their low toxicity, easy synthesis, and stability [19].

LDHs are two-dimensional anionic clay materials with the general formula [M^2+^_1-x_M^3+^_x_(OH)_2_]^x+^(A*^n^*^−^_x/n_). yH_2_O, where M^2+^ denotes divalent cations (Zn^2+^, Ni^2+^, Cu^2+^, etc.), M^+3^ denotes trivalent cations (Al^3+^, Fe^3+^, Cr^3+^, etc.), A*^n^*^−^ is an anion, and × = M^3+^/M^2+^ + M^3+^ with a value of 0.2 to 0.33 [19]. LDHs can be utilized in many different fields, including those involving adsorption, photocatalysis, catalysis, electrochemistry, and sensors, owing to their adjustable and layered structures, the composition of multiple metal species, and adjustable metal ratios [20]. To improve the catalytic activity of LDHs, their tunable structures are exploited so that they can be doped with metal elements [21]. The metal dopants can increase the number of active sites, catalytic stability, and electronic conductivity of LDHs [22]. For instance, Wani et al. [23] investigated the photodegradation of Rose Bengal (RB) in the presence of ZnAl LDH and Nd-doped ZnAl LDH under visible light irradiation. The degradation efficiency of ZnAl LDH (64 %) increased to 93 % by doping with neodymium (Nd) because Nd^3+^ ions aid the separation of electron-hole pairs. Another study found that the photocatalytic degradation efficiency of methylene blue in the presence of CuFe LDH was 41.56 %, while ZrRGO-doped CuFe LDH improved the degradation efficiency of methylene blue to 95.2 % by inducing in it a relatively quick charge transfer [24]. Furthermore, another study found that the photocatalytic degradation of 2,4 dinitrophenol NiFe LDH and S-doped graphitic carbon nitride with NiFe LDH (SGCN/NiFe LDH) exhibited 55 % and 98 % degradation efficiencies, respectively. In this case, the S dopant increased the number of active sites, and SGCN/NiFe LDH decreased the electron-hole recombination of the NiFe LDH [25].

In this study, a ZnFe LDH was doped with lanthanum (La) to improve its sonocatalytic properties. La has a special and active 4f electronic configuration that promotes electron transition and the production of new energy levels. La also improves catalytic performance by inhibiting electron-hole recombination owing to its electron scavenging property [26], [27]. The physicochemical properties of the LDH were determined using various characterization techniques. The sonocatalytic activities of the synthesized ZnFe LDH and La-doped ZnFe LDH alongside activated PDS were studied with respect to their metribuzin degradation. The influence of operating factors, such as catalyst dosage, pollutant concentration, PDS concentration, initial pH, light intensity, and the presence of radical scavengers and enhancers, on metribuzin degradation, was investigated. The reusability and stability of the La-doped ZnFe LDH catalyst were evaluated over four consecutive runs. The amount of metal leaching of the La-doped ZnFe LDH was evaluated using inductively coupled plasma-mass spectrometry (ICP-MS) analysis. In addition, the degradation performance of the synthesized sample was investigated in a wastewater matrix. Finally, the intermediates generated during the sonocatalytic process were determined using gas chromatography-mass spectrometry (GC–MS).

## 2 Materials and methods

### 2.1 Materials

Zinc chloride (ZnCl_2_), iron (III) chloride hexahydrate (FeCl_3_·6H_2_O, ≥ 99 %), lanthanum (III) chloride heptahydrate (LaCl_3_·7H_2_O), potassium peroxydisulfate (K_2_S_2_O_8_^2−^), hydrogen peroxide (H_2_O_2_, 30 %), isopropanol ((CH_3_)_2_CHOH, ≥ 99.5 %), sodium percarbonate (Na_2_CO_3_·1.5H_2_O_2_), 1,4-benzoquinone (C_6_H_4_O_2_, 99 %), formic acid (HCOOH), sodium hydroxide pellets (NaOH, 99 %), hydrochloric acid (HCI, 30 %), sodium nitrate (NaNO_3_, 99 %), nitric acid (HNO_3_, 65 %), diethyl ether ((C_2_H_5_)_2_O), N,O-Bis(trimethylsilyl)acetamide (C_8_H_21_NOSi_2_), and ethanol (C_2_H_5_OH, ≥99.5 %) were purchased from Merck, Germany and used in our study. A wastewater sample was obtained from the Gebze Organized Industrial Zone in Turkey.

### 2.2 Synthesis of La-doped ZnFe LDH

ZnFe LDH and La-doped ZnFe LDH were synthesized using the co-precipitation method [18]. The ZnFe LDH was synthesized using certain amounts of ZnCl_2_ and FeCl_3_·6H_2_O with a Zn: Fe molar ratio of 3:1 was dissolved in 120 mL deionized water purged with N_2_ gas. Then, under continuous stirring, a 2 mol/L NaOH solution was added dropwise until the pH of the solution reached 8. The mixture was then stirred for 24 h. Finally, the resulting suspension was centrifuged, washed several times with deionized water, and dried in an oven at 50 °C for 7 h to obtain the ZnFe LDH. For the preparation of the La-doped ZnFe LDH, the molar ratio of Zn, Fe, and La was adjusted to Zn:(Fe + La) = 3:1, and the procedure followed for the synthesis of the ZnFe LDH was followed for the synthesis of the La-doped ZnFe LDH.

### 2.3 Instrumentation

The crystal phase formation of the as-prepared samples was determined using an X-ray diffractometer (XRD) (Bruker D8 Advance, Germany) with Cu Kα radiation (0.15406 nm, 40 kV, 40 mA). The morphologies of the samples were examined by scanning electron microscope (SEM) (Philips XL30 SFEG, the Netherlands) and HRTEM analyses (JEOL JEM-2100 Plus, Japan, operated at 200 kV). The elemental compositions of the samples were determined by energy-dispersive X-ray spectroscopy (EDS) analysis (Philips XL30 SFEG, Netherlands). Fourier transform infrared (FT-IR) analysis (Perkin Elmer Spectrum 100, Germany) was performed to determine the functional groups of the samples. The chemical states of the sample were identified using X-ray photoelectron spectrometer (XPS) analysis (Thermo Scientific Escalab 250Xi+, USA). N_2_ adsorption–desorption analysis, using the BELSORP model of Mini II (Japan), was used to explore the samples’ specific surface area and pore structure. The differential reflectance spectroscopy (DRS) of the samples was recorded using a Shimadzu UV-2600 spectrophotometer (Japan). The leaching concentrations of lanthanum, zinc, and iron ions were determined by ICP-MS using an Agilent 7800 (USA). To determine the by-products generated during the sonocatalytic degradation of metribuzin, GC–MS analysis was performed using an Agilent 6890 N (USA).

### 2.4 Sonocatalytic activity tests

The sonocatalytic performances of La-doped ZnFe LDHs were evaluated based on their degradation of metribuzin. In a typical process, a certain amount of La-doped ZnFe LDH was added to 100 mL of metribuzin solution before sonication. The suspension was then sonicated in an ultrasonic bath (120 W, 45 kHz, USC300T, VWR, USA). A specific amount of PDS was added to the solution before beginning the experiment. At 10 min intervals, 5 mL of the sample was collected and then filtered with a 0.22 µm syringe filter. Finally, the metribuzin degradation efficiency was determined using a UV–Vis spectrophotometer (Perkin Elmer, Lambda 25, USA) at a maximum wavelength (λ_ma_x__) of 294 nm. Additionally, to better understand the synergy in the process, the synergy factor (SF) was calculated using **Eq.** (1)**.** [18].

(1) $$\mathrm{SF}=\;\frac{k_{app\left(\mathrm{US}/\;La-doped\;ZnFe\;LDH/\;PDS\right)}}{k_{app\left(\mathrm{US}\right)}+k_{app\left(\mathrm{La}-doped\;ZnFe\;LDH\right)}+k_{app\left(\mathrm{PDS}\right)}}$$

The calculation of “degradation turnover (dTON)” was developed to assess the activity of the degradation system [28]. In our study, the dTON was calculated using the following equation:

(2) $$\mathrm{dTON}\;=\frac{\left[C_{i}\right]\;-\;\left[C_{f}\right]}{t\;\times \;[A]\;}$$

where C_i_, C_f_, t, and A are the initial metribuzin concentration (µmol/L), final metribuzin concentration (µmol/L), reaction time (h), and catalyst concentration (g/L), respectively. It is also important to evaluate the suitability of processes from an economic perspective. The term “electrical energy per order (E_EO_)” was developed by Bolton et al. [29] to explain the amount of electrical energy required to treat a specific quantity of pollution in a given volume of solution. In our study, the E_EO_ of the US/La-doped ZnFe LDH/PDS process was determined using the following equation:

(3) $$E_{\mathrm{EO}}=\;\frac{P\;\times \;t\;\times \;1000}{V\;\times 60\times \;\log\;\left(C_{i}/C_{f}\right)}$$

where P, t, V, C_i_, and C_f_ are the US input power (kW), treatment time (min), reactor volume (L), initial pollutant concentration, and final pollutant concentration, respectively. Moreover, the sonocatalytic degradation of metribuzin in wastewater in the presence of La-doped ZnFe LDH was studied. This experiment was carried out for 360 min by adding 15 mg L^−1^ of metribuzin to wastewater with a total volume of 100 mL. All measurements were done at least in duplicate or triplicate, and average values with an error <2.5 % are reported.

## 3 Results and discussion

### 3.1 Materials characterization

**Fig. 1:**
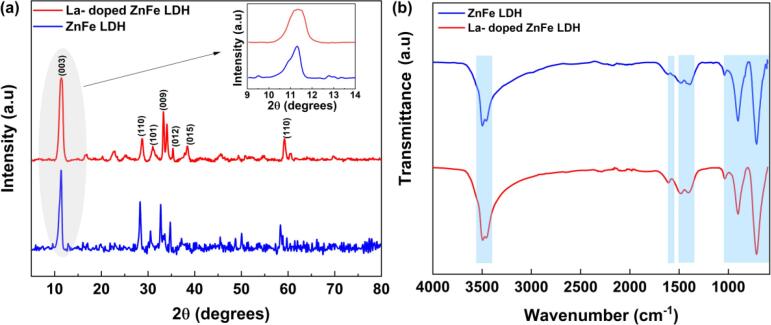
The XRD pattern (a) and FT-IR spectra of La-doped and undoped ZnFe LDHs (b).

The XRD patterns of the ZnFe LDH and La-doped ZnFe LDH samples are shown in Fig. 1**a.** The ZnFe LDH exhibited diffraction peaks corresponding to the (0 0 3), (1 1 0), (1 0 1), (0 0 9), (0 1 2), (0 1 5), and (1 1 0) planes centered at 2θ = 11.38°, 28.28°, 30.52°, 32.7°,34.76°, 37.16°, and 58.36°, respectively, which is in agreement with the JCPDS card no 38–0486 [28]. The presence of ZnFe LDH peaks in the pattern of doped LDH indicates that the LDH structure was preserved, and La atoms were successfully incorporated into the LDH structure. As can be seen in the magnified region, the position of the peak remained the same without a shift. This indicates that the La atoms occupied the octahedral sites in the brucite-like LDH layers instead of remaining as separate impurity phases or adsorbing on the surface. Similar results were reported for third metal-doped LDH materials [27]. However, the magnified peak became relatively broader after La doping, which suggests a reduction in the crystallinity of ZnFe LDH. The lattice parameters of ZnFe LDH and La-doped ZnFe LDH are presented in Table 1. The interplanar spacing of ZnFe LDH and La-doped ZnFe LDH was calculated by Bragg's law from (0 0 3) and (1 1 0) reflection; 2d_(hkl)_sinθ = nλ, where d_(hkl)_ is the spacing of the crystal layers, θ is the angle of diffraction n is diffraction order, and λ is the X-ray wavelength. Accordingly, the interplanar spacing of ZnFe LDH and La-doped ZnFe LDH was 7.76 Å and 7.88 Å, respectively. The parameter “a”, which represents the distance between the cations, is the same for both materials. The slight change in the “c” parameter, which indicates the distance between the layers, can be attributed to the water molecules between the layers [30].

**Table 1:** Structural parameters of the synthesized materials.

Samples	Lattice parameters			
	dhkl-spacing (003) (Å)	dhkl-spacing (110) (Å)	a(Å)	c (Å)
ZnFe LDH	7.76	1.57	3.14	23.28
La-doped ZnFe LDH	7.88	1.57	3.14	23.64

To determine their functional groups, the FT-IR spectra of the ZnFe LDH and La-doped ZnFe LDH samples were recorded (Fig. 1**b)**. The ZnFe LDH and La-doped ZnFe LDH showed similar spectra. The strong peaks observed at approximately 3455.5 cm^−1^ are attributed to the stretching vibration of hydrogen-bonded hydroxyl groups from both the hydroxyl group in the layers and the interlayer water [18]. The peaks observed at 1607.5 cm^−1^ are explained by the bending vibration of the interlayer water molecules (*v*_H_—_O_—_H_) [31]. 1350–1500 cm^−1^ due to the stretching vibration of carbonate anions in the interlayer [22]. The observed peaks lower than 1030.0 cm^−1^ are related to M−O and O–M–O stretching vibrations (M: Zn, Fe, or La [32]. However, it’s difficult to differentiate individual metal oxygens bonds because they usually appear as overlapped bands.

**Fig. 2:**
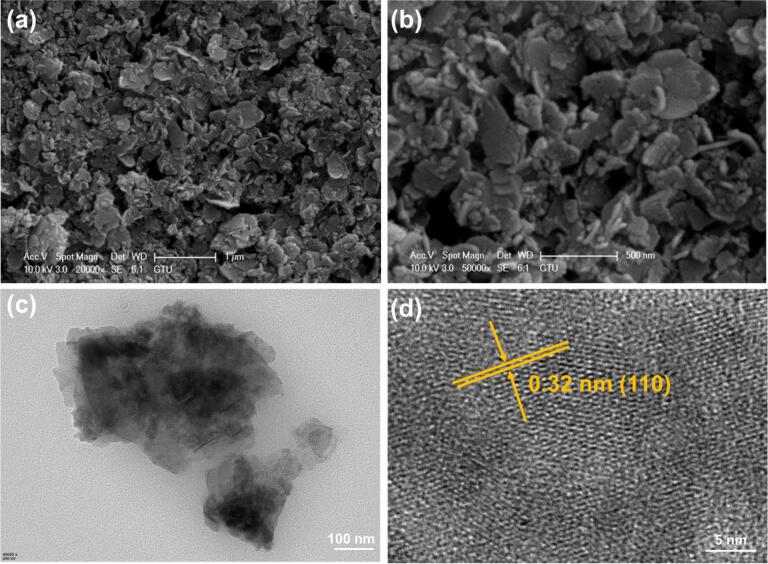
The SEM (a and b) and TEM (c and d) images La-doped ZnFe LDH.

SEM and TEM analyses were performed to identify the morphologies and microstructures of the catalysts, respectively. As shown in Fig. 2a and b, the La-doped ZnFe LDH exhibited a lamellar structure with an average thickness of 26 nm. The HRTEM images also indicated that the La-doped ZnFe LDH had a lamellar structure (Fig. 2**c and d**). The interplanar spacing was calculated as 0.32 nm, which corresponds to the (1 1 0) lattice plane of the LDH.

**Fig. 3:**
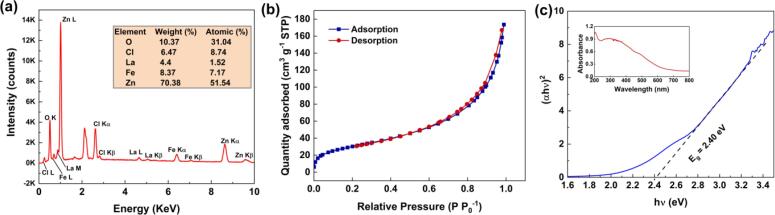
The EDS spectrum (a), N_2_ adsorption–desorption isotherm (b), and (αhʋ)^2^-hʋ curve of La-doped ZnFe LDH (inset shows the UV–vis diffuse reflectance) (c).

The presence of Zn, Fe, Cl, and La was confirmed by EDS analysis (Fig. 3**a**). Using ICP-OES analysis, the weighted of Zn, Fe, and La in the composition of La-doped ZnFe LDH were determined to be 81.96 %, 12.72 %, and 5.31 %, respectively. The N_2_ adsorption–desorption isotherms of the synthesized La-doped ZnFe LDH are illustrated in Fig. 3**b.** According to the IUPAC classification, the synthesized LDH showed type IV isotherms, indicating that the samples had a mesoporous structure. The BET surface area (S_BET_), pore volume (V_m_), and average pore diameter of the La-doped ZnFe LDH were determined as 110.93 m^2^ g^−1^, 0.27 cm^3^ g^−1^, and 9.67 nm, respectively. Previous studies have reported a range of 46.9–108.8 m^2^ g^−1^ for the S_BET_ of ZnFe LDH [33], [34], [35]. La doping can enhance the contact areas of LDH by increasing the surface roughness of LDH [27]. The fact that La-doped ZnFe LDH has a higher S_BET_ than ZnFe LDHs can provide greater degradation efficiency.

The light absorption capacity of the La-doped ZnFe LDH was determined using its UV–vis DRS spectrum. As shown in Fig. 3**c**, the adsorption peaks of the La-doped ZnFe LDH appeared in the UV wavelength region (approximately 300 nm). The bandgap energy (E_g_) of the La-doped ZnFe LDH was determined using the Kubelka-Munk formula [28], and the curve of (αhʋ)^2^ against (hʋ) is illustrated in Fig. 3**c.** The E_g_ value of the La-doped ZnFe LDH was determined to be 2.40 eV.

**Fig. 4:**
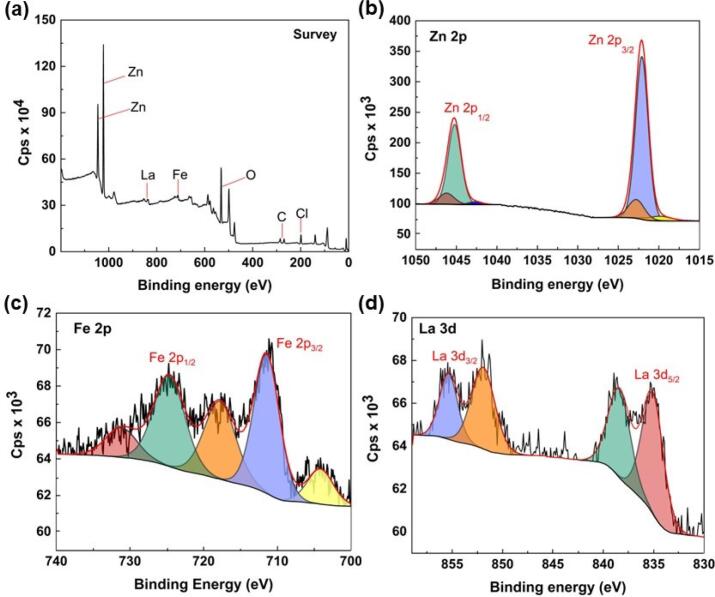
Survey spectrum for La-doped ZnFe LDH (a) and high-resolution XPS spectra of Zn 2p (b), and La 3d and (d) of La-doped ZnFe LDH.

XPS analysis was conducted to determine the surface elemental state of the La-doped ZnFe LDH. The XPS survey spectrum showed the presence of Zn, O, Fe, C, Cl, and La in the catalyst’s structure **(**Fig. 4**a).** The high-resolution Zn 2p spectra **(**Fig. 4**b)**, which showed two distinct peaks at 1022.10 eV (Zn 2p_3/2_) and 1045.30 eV (Zn 2p_1/2_), confirmed the existence of Zn species in the divalent oxidation state [33]. Fe 2p_3/2_ and Fe 2p_1/2_ were found to have binding energies of 711.10 eV and 724.70 eV, respectively, based on the high-resolution spectra of Fe 2p [36]. In addition, three satellite peaks at 704.40 eV, 717.60 eV, and 731.70 eV appeared **(**Fig. 4**c)** [37]. As illustrated in Fig. S1, the peaks that appeared at 198.60 eV and 200.10 eV binding energies can be attributed to Cl 2p_3/2_ and Cl 2p_1/2_, respectively [38]. In the La 3d spectrum, the peaks at 835.30 eV and 852 eV were related to La 3d_5/2_ and La 3d_3/2_, respectively [39]. Moreover, two satellite peaks at 838.90 eV and 855.40 eV were associated with La 3d_5/2_ and La 3d_3/2_, respectively [40]. According to the deconvoluted La 3d spectrum, the La element stays in the La^3+^ form in the LDH structure. Simultaneously, while the doped La^3+^ ions replace part of the Fe^3+^, a _d_-electron transition takes place due to the intrinsic electronic structure of La^3+^ [21]. This changes the spatial structure of LDH and creates structural defects resulting in an improvement in the catalytic activity.

### 3.2 The effect of operational parameters

**Fig. 5:**
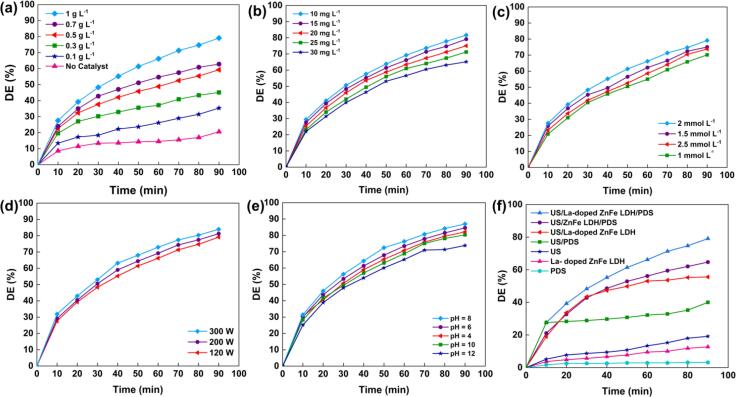
The effect of La-doped ZnFe LDH dosage (a), metribuzin concentration (b), PDS concentration (c), US power (d), initial pH (e), and different processes (f) (Experimental condition: [Metribuzin]_0_ = 15 mg/L, [La doped ZnFe LDH] = 1 g/L, [PDS] = 2 mmol/L, pH = 6.35 (natural), and US power = 120 W).

The influence of the La-doped ZnFe LDH dosage (0.1–1 g/L) on the metribuzin degradation efficiency is illustrated in Fig. 5**a**. The degradation efficiency increased from 35.9 % to 79.1 % by increasing the catalyst dosage from 0.1 g/L up to 1 g/L. This can be explained by the fact that as the catalyst dosage increased, more active sites were available, which facilitates the formation of reactive species. Similar results have been reported previously [41]. Accordingly, the optimum catalyst dosage was determined to be 1 g/L for the remainder of the experiments.

The effect of the initial metribuzin concentration on the degradation efficiency was also investigated (Fig. 5**b**). Our results indicate that the highest degradation efficiency was obtained at 10 mg L^−1^. However, the efficiency gradually decreased from 81.6 % to 65.1 % with the increase in pollutant concentration from 10 to 30 mg L^−1^. This reduction can be explained by the fact that the surfaces of the catalysts are completely occupied by metribuzin molecules. Additionally, at higher concentrations of pollutants under constant conditions, limited amounts of ROS are produced, which is insufficient for complete degradation [18].

Owing to the creation of a synergistic effect with US, the concentration of PDS plays a significant role in the degradation efficiency of metribuzin. The effect of the PDS concentration (1–2.5 mmol/L) on degradation was investigated. As shown in Fig. 5**c**, the degradation efficiency of metribuzin increased from 70.2 % to 79.1 % when the PDS concentration increased from 1 to 2 mmol/L. This can be explained by the increased formation of sulfate radicals and the consequent increase in LDH catalytic activity with higher PDS concentration. However, the degradation efficiency decreased to 73.8 % when the PDS dose was increased to 2.5 mmol/L. This could be related to the quenching reactions of SO_4_^•−^ and ^•^OH radicals at high PDS dosages **(Eqs. (4–6))** [42]. Therefore, the optimum PDS concentration was determined to be 2 mmol/L.

(4) $$SO_{4}^{∙-}+S_{2}O_{8}^{2-}\to \;SO_{4}^{2-}+S_{2}O_{8}^{∙-}\;$$

(5) $$SO_{4}^{∙-}+SO_{4}^{∙-}\to S_{2}O_{8}^{2-}$$

(6) $$S_{2}O_{8}^{2-}+{\;}^{∙}OH\to \;S_{2}O_{8}^{∙-}+\;OH^{-}$$

One of the parameters that greatly influences degradation efficiency is US power, which affects the number of collapsing bubbles produced [43]. Fig. 5**d** shows the sonocatalytic degradation of metribuzin using ultrasonic irradiation powers of 120 W, 200 W, and 300 W. As seen in the figure, the degradation efficiency increased slightly with increasing US power. The maximum degradation efficiency was 83.9 % at 300 W. This can be attributed to the increased generation of ROS and facilitation of the formation of sulfate radicals by the dissociation of PDS molecules. Additionally, the turbulence of the system also increases with an increase in the US power, which has an impact on the mass transfer rate [18]. Because there was no significant difference in the degradation efficiency with increasing US power and to reduce the energy consumption, 120 W was selected as the optimum US power.

The effect of initial pH metribuzin solution ranging from 4 to 12 on metribuzin degradation is illustrated in Fig. 5**e**. The highest degradation was observed at pH = 8, and the degradation ability was reduced under alkaline conditions. To explain this trend, the pH at the point of zero charge (pH_pzc_) of La-doped ZnFe LDH was calculated **(**Fig. S2**)**. The pH_pzc_ of the sonocatalyst was 7.1, and thus, the surfaces of the catalyst were positively and negatively charged at pH < 7.1 and pH > 7.1, respectively [43]. Moreover, the reported pKa value of metribuzin is 1.0 [44]. Thus, metribuzin molecules are negatively charged at a pH above 1 and positively charged at pH values below 1. Because both the catalyst surface and metribuzin molecules are negatively charged above a pH of 7.1, electrostatic repulsion between them results in a reduction in the degradation efficiency [43]. Additionally, the decreased efficiency may be related to the conversion of SO_4_^•−^ to ^•^OH radicals, which have a lower oxidation potential under alkaline conditions [45].

Considering the information obtained from the effect of parameters, the following experimental conditions were selected as the optimal conditions to continue further experiments: 15 mg L^−1^ of metribuzin, La-doped ZnFe LDH concentration of 1 g/L, PDS concentration of 2 mmol/L, pH of 6.35, and US power of 120 W.

### 3.3 Degradation of metribuzin by different processes

**Fig. 6:**
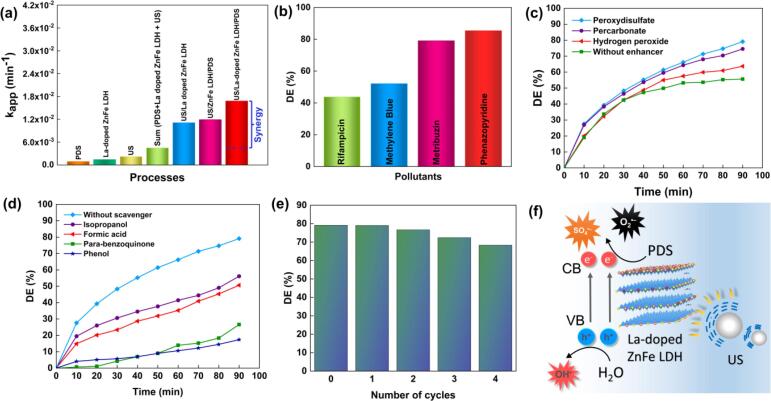
The effect of synergy factor (a), different pollutants (b), enhancers (c), scavengers (d), reusability behavior (e), and proposed degradation mechanisms of metribuzin by US/La-doped ZnFe LDH/PDS process (f). (Experimental condition: [Metribuzin]_0_ = 15 mg/L, [La doped ZnFe LDH] = 1 g/L, pH = 6.35 (natural), and US power = 120 W).

The degradation of metribuzin was examined through various processes, such as adsorption (La-doped ZnFe LDH), ultrasonication (US), PDS, US/La-doped ZnFe LDH, and US/La-doped ZnFe LDH/PDS (Fig. 5**f**). In addition, degradation activities in the presence of PDS (US/ZnFe LDH/PDS and US/La-doped ZnFe LDH/PDS) were compared. Because PDS was not activated by the US or the catalyst, PDS alone degraded 3.2 % of metribuzin. The adsorption of the pollutant molecules on the surface of La-doped ZnFe LDH is 12.7 %. The degradation efficiency of the US was only 19.1 % owing to the limited ^•^OH radicals generation. Due to the high ^•^OH radicals generated by the US/La-doped ZnFe LDH process, the degradation efficiency increased to 55.6 %. Degradation efficiencies of 64.8 % and 79.1 % were obtained by US/ZnFe LDH/PDS and US/La-doped ZnFe LDH/PDS processes, respectively. This result can be explained by the fact that doping with La confers a high specific surface area, which increases the number of active sites. Additionally, a pseudo-first-order kinetic model was applied to calculate the reaction rate constant (k_app_). As shown in Fig. 6**a**, the processes had low k_app_ values when applied individually. The synergistic effect of the catalyst, US, and PDS was revealed by the fact that the k_app_ value of the US/La-doped ZnFe LDH/PDS process was greater than the sum of the k_app_ values of the separate processes.

To understand the synergistic effect of the process better, the synergy factor was calculated using **Eq.** (1). The synergy factor of the process was found to be 3.73, which revealed a significant synergistic effect between the US, La-doped ZnFe LDH, and PDS. Moreover, the degradation activity of the La-doped ZnFe LDH was evaluated in terms of dTON parameters. The dTON of the process was calculated as 37.5 µmol h^−1^ g_cat_^−1^. The E_EO_ value of the process was calculated as 2537.6 kWh m^−3^ order ^− 1^ using **Eq.** (3). In comparison, the sonocatalytic degradation of ofloxacin by TiO_2_ and ZnO were reported to have an E_EO_ of 7040.6 and 6154.5 kWh m^−3^ order ^–1^, respectively [46]. This is because introducing of PDS into the process caused an increase in degradation, which reduced energy consumption.

Additionally, the effectiveness of the PDS-assisted sonocatalytic process in degrading other pollutants, such as rifampicin (RIF), methylene blue (MB), and phenazopyridine (PHP) was examined. As shown in Fig. 6**b**, the degradation efficiencies of RIF, MB, and PHP were 43.83, 52.13, and 85.4 %, respectively.The dTON parameter was also calculated as 6.33, 12, and 45.8 µmol h^−1^ g_cat_^−1^ for RIF, MB, and PHP, respectively, depending on the activity of the degradation mechanism.

Selected pollutants are members of different classes and consist of diverse molecular groups. Therefore, the difference in their degradation efficiencies could be attributed to their molecular structures and chemical compositions. The lower removal efficiency of RIF can be explained by its stable molecular structure that is mainly composed of a heterocyclic ring containing naphthoquinone and much higher molecular weight than other pollutants (MW_RIF_: 822.94 g mol^−1^). MB (MW_MB_: 319.85 g mol^−1^) is composed of two aromatic rings and a central heterocyclic ring called thiazine, whereas metribuzin only contains one heterocyclic ring and has a much lower molecular weight (MW_Metribuzin_: 214.29 g mol^−1^) than MB. This could be the reason for the greater removal efficiency of metribuzin than MB. PHP, which has the smallest molecule size (MW_PHP_: 213.24 g mol^−1^), has been reported to degrade mostly by the breaking of the N=N bond by ROS [47]. Therefore, US/La-doped ZnFe LDH/PDS proved to be efficient in the degradation of pharmaceuticals and dyes, in addition to herbicides.

### 3.4 Effect of enhancers and scavengers

The impact of oxidants, including peroxydisulfate, percarbonate, and hydrogen peroxide, on the sonocatalytic degradation of metribuzin was investigated, and the results are presented in Fig. 6**c**. In the presence of hydrogen peroxide, the degradation efficiency only increased from 55.6 % to 63.7 %. ^•^OH radicals were formed as a result of the dissolution of H_2_O_2_ according to the following reactions [48]:

(7) $$H_{2}O_{2}+ultrasonic\;irradiation\;\to \;2^{\;∙}OH\;\;$$

(8) $$H_{2}O_{2}+\;e^{-}\to {\;}^{∙}OH+\;OH^{-}\;$$

According to the following reactions, the addition of percarbonate enhanced the degradation efficiency of metribuzin from 55.6 % to 74.5 % by producing additional ^•^OH radicals following **Eqs.** (9), (7), respectively [49].

(9) $$2Na_{2}CO_{3}\cdot 3H_{2}O_{2}\to \;2Na_{2}CO_{3}+\;3H_{2}O_{2}$$

The sonocatalytic degradation increased from 55.6 % to 79.1 % by adding PDS. The enhancing ability of PDS was greater than that of other oxidant agents. This was probably related to the decomposition of PDS into SO_4_^•−^ and the formation of additional ^•^OH radicals, as described in **Eqs.** (10), (11) [50]:

(10) $$S_{2}O_{8}^{2-}+ultrasonic\;irradiation\to 2SO_{4}^{∙-}$$

(11) $$SO_{4}^{∙-}+H_{2}O\;\to \;H^{+}+\;SO_{4}^{2-}{+}^{\;∙}OH\;$$

To determine the ROS that contributed to the sonocatalytic process, isopropanol (^•^OH scavenger), phenol (^•^OH and SO_4_^•−^ scavenger), formic acid (h_VB_^+^ scavenger), and *p*-benzoquinone (O_2_^•−^ scavenger) were added to the solution. The presence of isopropanol, formic acid, p-benzoquinone, and phenol resulted in a decline in the degradation efficiency from 79.1 % to 56.1, 50.6, 26.6, and 17.3 %, respectively (Fig. 6**d**). These results indicate that SO_4_^•−^ and superoxide radicals (O_2_^•−^) played important roles in the metribuzin degradation through sonocatalysis. Moreover, while the degradation efficiency decreased by 23 % in the presence of isopropanol and by 61.8 % in the presence of phenol, the effect of sulfate radical production can be inferred from the difference between these two scavengers. Both SO_4_^•−^ and ^•^OH radicals were involved in the degradation process, but as phenol had a stronger inhibitory effect than isopropanol, it can be concluded that SO_4_^•−^ radicals were the main oxidant species in the US/La-doped ZnFe LDH/PDS process [51]. Similar results have been reported previously [45], [50], [51].

### 3.5 Reusability of the catalyst

When considering the effectiveness of a catalyst in long-term applications and from an economic perspective, stability and reusability are crucial factors [52]. Four consecutive cycles were performed under the same conditions to identify the reusability of the La-doped ZnFe LDH. After the sonocatalytic process, the La-doped ZnFe LDH was collected, washed with distilled water, and dried. As shown in Fig. 6**e**, La-doped ZnFe LDH exhibited 68.3 % degradation efficiency of metribuzin after the fourth cycle, indicating remarkable reusability. The slight reduction in efficiency can be attributed to metribuzin and its intermediates occupying the catalyst surface, which results in a reduction in the number of active sites and the inhibition of catalytic performance [53]. The XRD patterns before and after the reuse experiments were compared to better understand the stability of the catalysts **(**Fig. S3**a)**. The results show that the surface composition of the catalyst did not change noticeably. SEM analysis showed that the La-doped ZnFe LDH mostly maintained its morphological structure (Fig. S3**b).** Additionally, a metal-ion leaching study was conducted to evaluate the stability of the catalyst. The ICP-OES analysis revealed that Zn had only leached 0.003 %, and no La or Fe leachings were detected. Thus, it can be concluded that the as-synthesized catalyst had high durability and stability.

### 3.6 Possible degradation mechanism by US/La-doped ZnFe LDH/PDS

Based on our findings, we suggest the following as a potential mechanism for the US/La-doped ZnFe LDH/PDS process **(**Fig. 6**f)**. First, cavitation bubbles went through a process of formation, growth, and collapse under ultrasonic irradiation. The collapse of cavitation bubbles causes localized hot spots, which can thermally decompose H_2_O into ^•^OH radicals according to **Eq.** (12) [13]. In addition, ultrasonic irradiation induces the generation of sonoluminescence e^−^/h^+^ pairs on the surface of the La-doped ZnFe LDH **(Eq.** (13)**)** [54]. Similarly, the chemical bonds in PDS are also broken by US irradiation, resulting in the production of SO_4_^•−^ radicals **(Eq.** (10)**)** [17]. The electrons produced (e^−^) interact with PDS and oxygen molecules to produce SO_4_^•−^ and O_2_^•−^, respectively **(Eqs. (14**–**15))** [50]**.** Meanwhile**,** the holes (h^+^) react with water molecules and hydroxyl anions to generate ^•^OH **(Eqs (16**–**17))** [18]. Finally, h^+^, O_2_^•−^, ^•^OH, and SO_4_^•−^ participate in the degradation of metribuzin and the intermediate products, according to **Eqs.** (18) [17].

(12) $$H_{2}O\;+US\;\;\to {\;}^{∙}OH+{\;}^{∙}H$$

(13) $$\mathrm{La}-doped\;ZnFe\;LDH+US\;\to \;La-doped\;ZnFe\;LDH\;(e^{-}+h^{+})$$

(14) $$S_{2}O_{8}^{2-}+e^{-}\to SO_{4}^{∙-}+SO_{4}^{2-}$$

(15) $$e^{-}+O_{2}\to O_{2}^{∙-}$$

(16) $$h^{+}+H_{2}O\;\to {\;}^{∙}OH+H^{+}\;$$

(17) $$h^{+}+OH^{-}\to {\;}^{∙}OH$$

(18) $$Reactive\;species\;(h^{+},O_{2}^{∙-},{\;}^{∙}OH,SO_{4}^{∙-})+Metribuzin\;\to degradation\;products$$

### 3.7 Degradation of metribuzin in real wastewater and generated intermediates

To assess the practical applicability of La-doped ZnFe LDH, the sonocatalytic degradation of metribuzin was performed in real wastewater. The supplied wastewater had the following characteristics: pH = 7.75, conductivity = 1905 µs cm^−1^, and TOC = 16 mg L^−1^. Fig. S4 shows the change in the UV–vis spectra of the sonocatalytic degradation of metribuzin by the La-doped ZnFe LDH in the real wastewater matrix. The degradation efficiency of metribuzin in wastewater reached 52.8 % after 360 min. The decrease observed in the degradation efficiency of wastewater compared to that in pure water can be attributed to the presence of organic matter and inorganic anions in real wastewater [55]. These materials have an inhibitory effect on the efficiency of degradation since they quench ROSs.[43]. However, the results clearly show that the La-doped ZnFe LDH has a successful performance in removing metribuzin from wastewater. Moreover, the dTON and E_EO_ parameters for metribuzin degradation in wastewater were calculated as 11.7 µmol h^−1^ gcat^−1^ and 21485.6 kWh m^−3^ order ^– 1^, respectively.

A GC–MS analysis was performed to determine the by-products of metribuzin degradation generated during the PDS-assisted sonocatalytic process. According to the structure of metribuzin, the degradation should first start with desulfurization and deamination [5], [56]. The degradation of metribuzin occurs mainly as a result of the breaking of the C—C, C—N, C—S, and N—N bonds [57]. The reactive species, SO_4_^−•^ and O_2_^•−^, that are produced during the process are responsible for the degradation. Based on the identified intermediates, the cleavage of the triazine ring results in the formation of linear compounds with mostly no more than four carbon atoms as the final degradation products of metribuzin. As shown in Table S1, six intermediates were identified during the ultrasonic degradation of metribuzin in the presence of PDS and La-doped ZnFe LDH. Some other molecules may exist in the reaction media that could not be detected owing to instrument limitations.

The efficiency of the US/La-doped ZnFe LDH/PDS process was compared with other catalytic degradation results **(**Table 2**).** The higher performances of some studies are because their applied US power or PDS dosages are greater than our study. Nevertheless, US/La-doped ZnFe LDH/PDS achieved comparable or higher performance with much lower power and chemical consumption, which shows the cost-effectiveness of the process.

**Table 2:** Comparison of sonocatalytic performances for different catalysts.

Catalyst	Process	Pollutant	Operational conditions	DE (%)	Ref.
CuZnTi-LDH	US	Methylene blue	[Methylene blue]:10 mg L^−1^, [CuZnTi-LDH]: 0.5 g/L, pH: 5.5 US power: N/A, and reaction time: 90 min.	71	[10]
CaMoO_4_	US	Acid Orange 7	[Acid Orange 7]: 15 mg L^−1^, [CaMoO_4_]: 1 g/L, pH:7, US power: 200 W, and reaction time: 120 min.	68	[13]
nZVI	US/PDS	Nonylphenol	[Nonylphenol]: 20 mg L^−1^, [nZVI]: 0.6 g/L, PDS:6.5 mmol/L, pH: 4.2, US power: 300 W, and reaction time: 50 min.	85	[45]
ZnO	US/PDS	Norfloxacin	[Norfloxacin]: 2 mg L^−1^, [ZnO]: 0.3 g/L, [PDS]:0.1 g/L, pH: 7.5, US power:200 W, and reaction time: 80 min.	66.8	[58]
Ag_3_PO_4_/CoWO_4_	US	Tetracycline	[Tetracycline]: 15 mg L^−1^, [Ag_3_PO_4_/CoWO_4_]: 1 g/L, pH: N/A, US power: 500 W, and reaction time: 90 min.	75.3	[59]
La- doped ZnFe LDH	US/PDS	Metribuzin	[Metribuzin]: 15 mg L^−1^, [La-doped ZnFe LDH]: 1 g/L, [PDS]: 2 mmol L^−1^, pH: 6.35, US power: 120 W, and reaction time: 90 min.	79.1	This work

## 4 Conclusion

In this study, La-doped ZnFe LDH was successfully synthesized (as shown by the XRD analysis results) using the co-precipitation method, and its sonocatalytic activity for the degradation of metribuzin through PDS activation was evaluated. The high specific surface area (110.93 m^2^ g^−1^) of La-doped ZnFe LDH provided high active sites for the sonocatalytic process. The US/La-doped ZnFe LDH/PDS (79.1 %) exhibited higher degradation efficiency compared to US/ZnFe LDH/PDS (64.8 %) and US/La-doped ZnFe LDH (55.6 %) under the optimum conditions of 1 g L^−1^ of catalyst dosage, 15 mg L^−1^ metribuzin, 2 mmol/L PDS, initial pH 6.35, and 120 W US power during the 90 min-long process. The synergy factor of the US/La-doped ZnFe/PDS process was determined to be 3.73, demonstrating the synergistic interaction between the US, La-doped ZnFe LDH, and PDS. In the presence of scavengers such as isopropanol, formic acid, p-benzoquinone, and phenol, the degradation efficiency declined from 79.1 to 56.1, 50.6, 26.6, and 17.3 %, respectively. The results revealed that SO_4_^•−^ radicals played a major role in the sonocatalytic degradation of metribuzin. In contrast, with the addition of other enhancers such as hydrogen peroxide and percarbonate, the degradation efficiency was increased to 63.7 % and 74.5 %, respectively. After four cycles, the degradation efficiency only decreased approximately by 11 %, indicating the high reusability of the catalyst. Finally, the degradation of metribuzin in real wastewater was explored, in which the degradation efficiency of the US/La-doped ZnFe LDH/PDS reached 52.8 % during 360 min of reaction time. A GC–MS analysis revealed six by-products of metribuzin degradation. In conclusion, we believe that our findings show that La-doped ZnFe LDH can be an innovative sonocatalyst for the degradation of herbicides from water and wastewater.

## CRediT authorship contribution statement

**Sultan Akdağ:** Investigation, Writing – original draft. **Tannaz Sadeghi Rad:** Writing – review & editing. **Ramazan Keyikoğlu:** Writing – review & editing. **Yasin Orooji:** Writing – review & editing. **Yeojoon Yoon:** Writing – review & editing. **Alireza Khataee:** Supervision, Writing – review & editing, Resources.

## Declaration of Competing Interest

The authors declare that they have no known competing financial interests or personal relationships that could have appeared to influence the work reported in this paper.

## Data Availability

No data was used for the research described in the article.
