# Supplementary material for: Peroxydisulfate-assisted sonocatalytic degradation of metribuzin by La-doped ZnFe layered double hydroxide
Source: Ultrason Sonochem. 2022 Nov 23;91:106236. doi: 10.1016/j.ultsonch.2022.106236 (PMC9709225; doi:10.1016/j.ultsonch.2022.106236)
Supplement: Supplementary data 1 [file mmc1.docx]

**Supporting Information**

**Peroxydisulfate-assisted sonocatalytic degradation of metribuzin by La-doped ZnFe layered double hydroxide**

**Sultan Akdağ ^a^, Tannaz Sadeghi Rad ^a^, Ramazan Keyikoğlu ^a,b^, Yasin Orooji^c^, Yeojoon Yoon,^d,*^ Alireza Khataee ^a,e,*^**

^a^ Department of Environmental Engineering, Faculty of Engineering, Gebze Technical University, 41400 Gebze, Turkey

^b^ Department of Environmental Engineering, Faculty of Engineering and Natural Sciences, Bursa Technical University, 16310 Bursa, Turkey

^c^ College of Geography and Environmental Sciences, Zhejiang Normal University, Jinhua 321004, China

^d^ Department of Environmental and Energy Engineering, Yonsei University, Wonju, Republic of Korea

^e^ Research Laboratory of Advanced Water and Wastewater Treatment Processes, Department of Applied Chemistry, Faculty of Chemistry, University of Tabriz, 51666−16471 Tabriz, Iran

*Corresponding authors:

akhataee@gtu.edu.tr

yajoony@yonsei.ac.kr


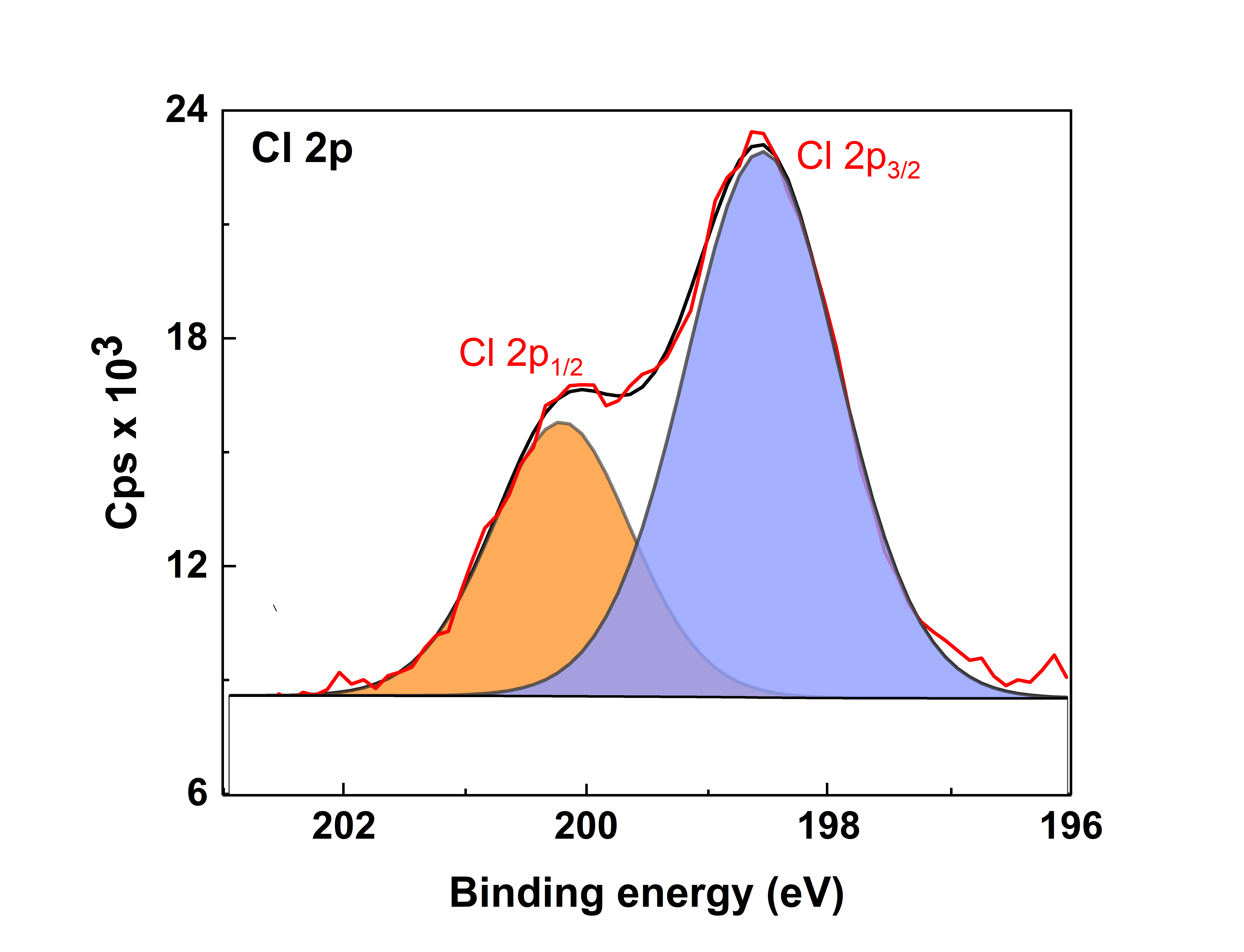


**Fig. S1.** High-resolution XPS spectra of Cl 2p .


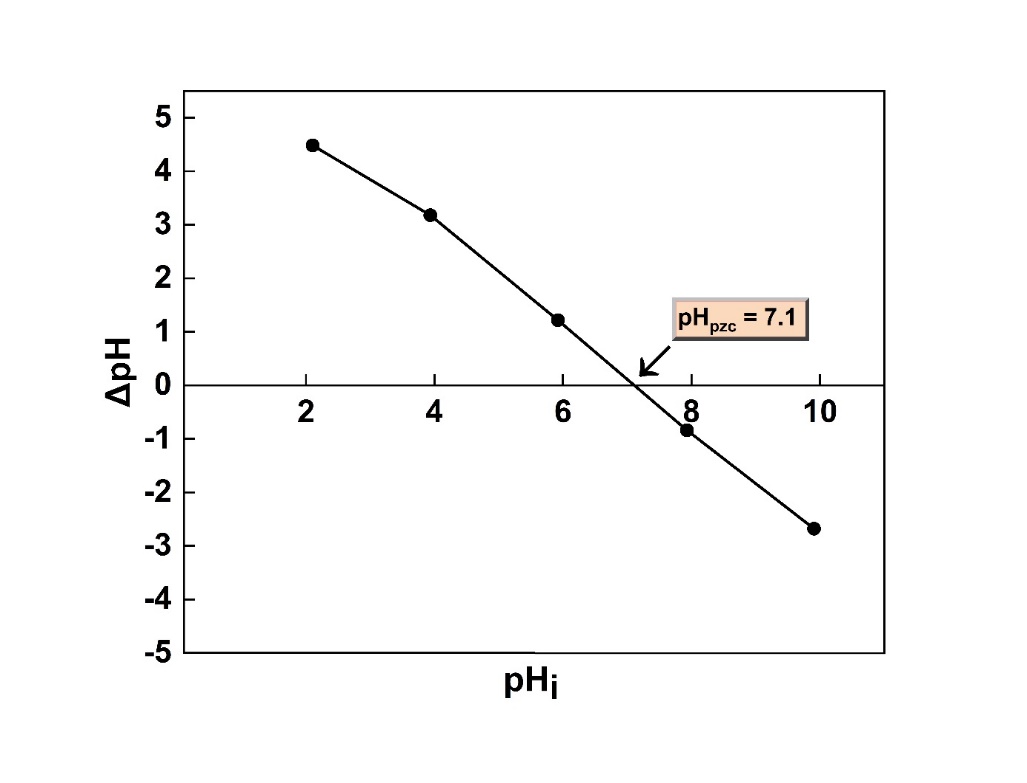


**Fig. S2.** The determination of pH_pzc_ of La-doped ZnFe LDH


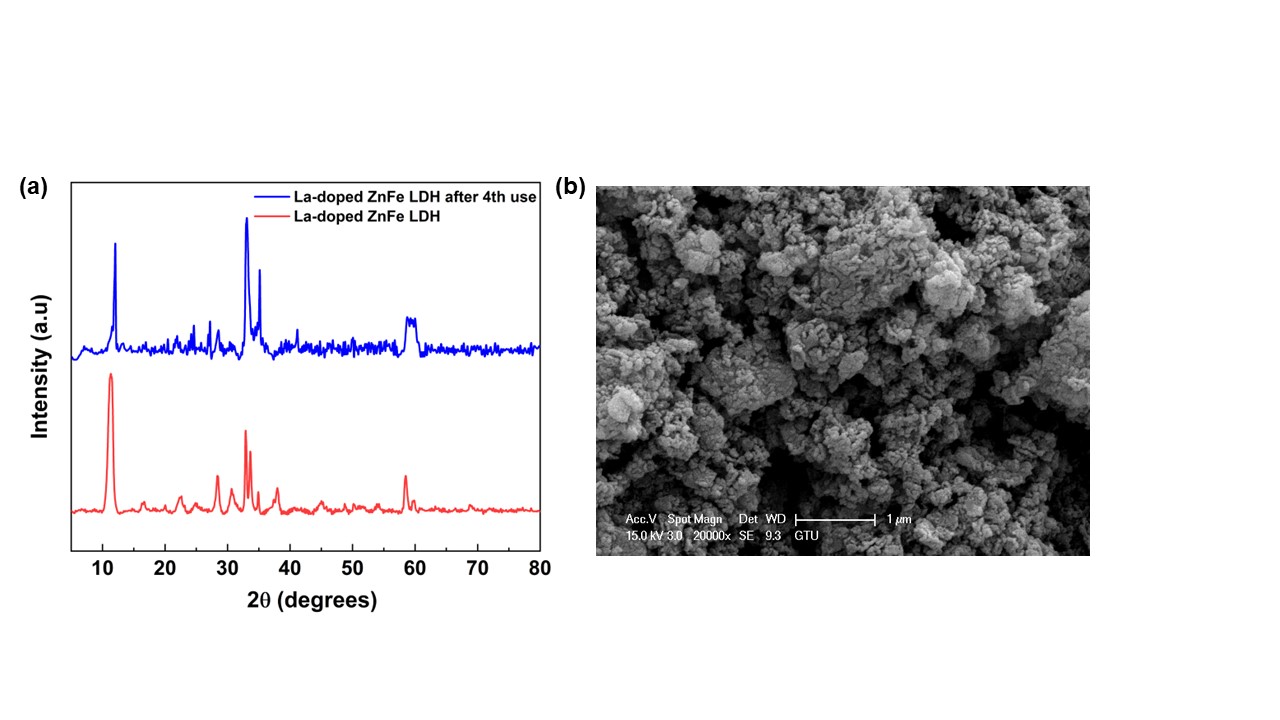


**Fig. S3**. The XRD patterns of La-doped ZnFe LDH before and after four consecutive runs (a), the SEM image of the used La-doped ZnFe LDH.


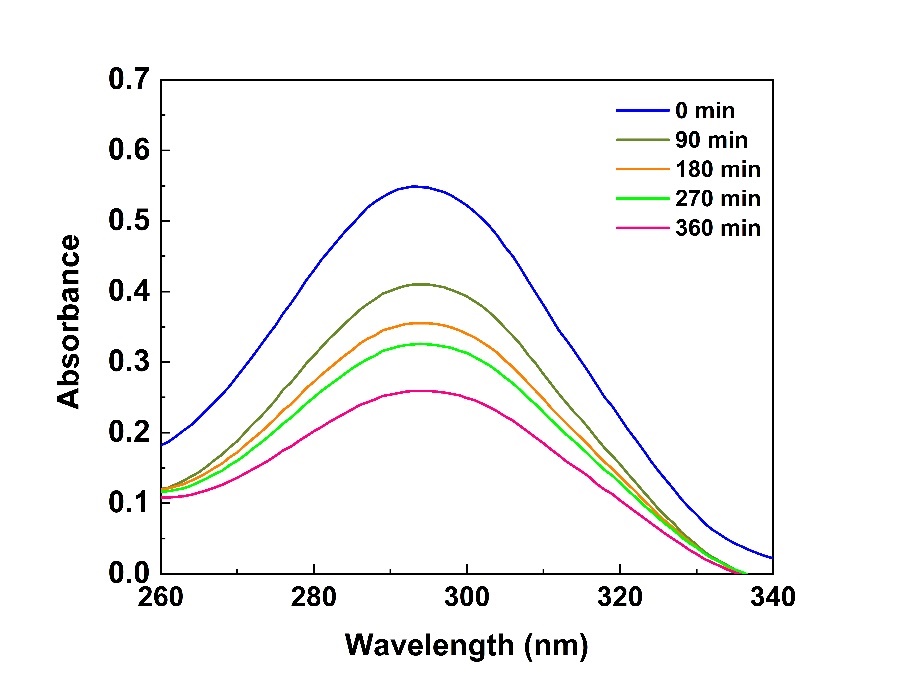


**Fig. S4.** The UV-Vis spectra of metribuzin at different times (Experimental conditions: [Metribuzin]_0_ = 15 mg L^-1^, [La doped ZnFe LDH] = 1 g L^-1^, [PDS] = 2 mmol L^-1^, US power = 120 W, and treatment time= 360 min).

**Table S1.** Generated intermediates during the sonocatalytic degradation of metribuzin. ([Metribuzin]_0_ = 15 mg L^-1^, [La doped ZnFe LDH] = 1 g L^-1^, [PDS]= 2 mmol L^-1^, pH= 6.35 (natural), and US power = 120 W).

| No. | Compound Names | Structures | t_R_(min) | Main fragments (m/z) /(percent) |
| --- | --- | --- | --- | --- |
| 1 | Acetamide | 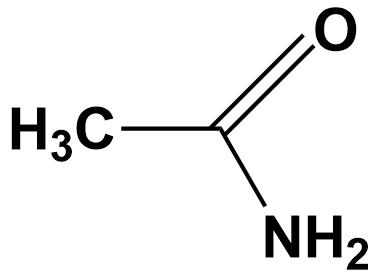 | 5.603 | 75.10(100%), 116.10(84.12%), 73.10(13.60%), 117.10(8.32%), 76.10(7.35%) |
| 2 | Ethanimidic acid | 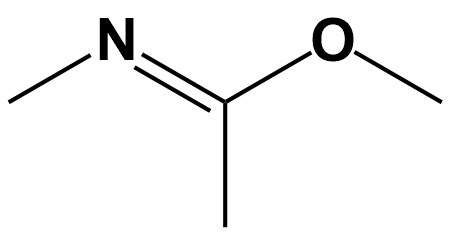 | 6.209 | 147.20(100%), 73.10(52.66%),148.05(21.02%), 203.20(19.09%), 188.10(11.75%) |
| 3 | Ethanamine | 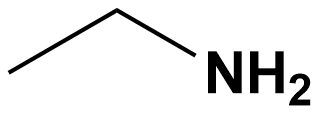 | 6.244 | 174.10(100%), 100.10(58.29%), 73.10(31.22%), 175.10(18.67%), 59.00(17.23%) |
| 4 | Alanine | 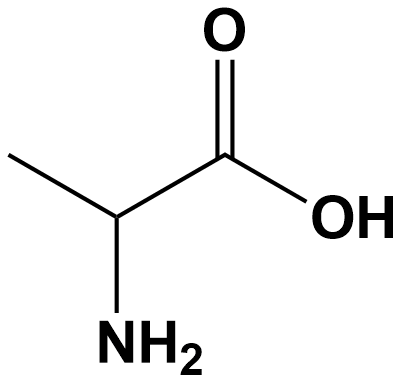 | 6.307 | 144.10(100%), 73.10(58.62%), 158.20(34.41%), 59.10(13.19%), 145.05(13.14%) |
| 5 | L-norvaline | 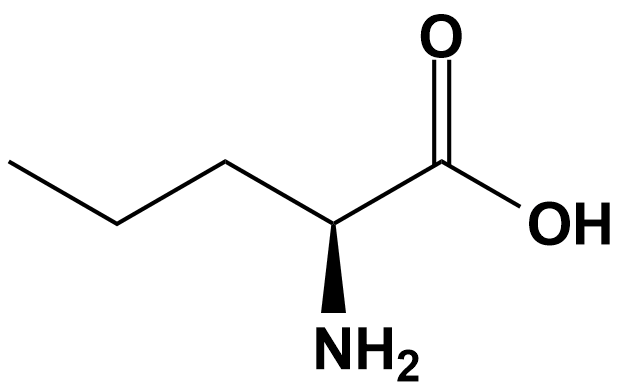 | 6.312 | 144.10(100%), 73.10(56.34%), 158.10(34.14%), 59.00(13.23%), 145.20(12.57%) |
| 6 | 2-Propenoic acid | 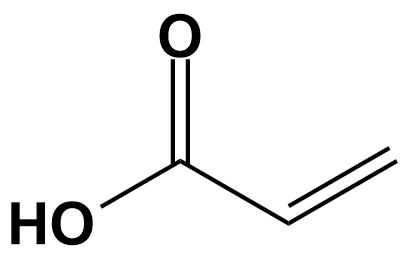 | 6.976 | 147.10(100%), 73.00(76.74%), 217.20(21.54%), 188.10(20.98%), 207.00(14.45%) |
